# Efficiency of advanced wastewater treatment technologies for the reduction of hormonal activity in effluents and connected surface water bodies by means of vitellogenin analyses in rainbow trout (*Oncorhynchus mykiss*) and brown trout (*Salmo trutta f. fario*)

**DOI:** 10.1186/s12302-015-0056-3

**Published:** 2015-09-30

**Authors:** Anja Henneberg, Rita Triebskorn

**Affiliations:** Animal Physiological Ecology, University of Tübingen, Auf der Morgenstelle 5, 72076 Tübingen, Germany

**Keywords:** Endocrine disruption, Micropollutants, Wastewater treatment plant, Fish, Vitellogenin

## Abstract

Endocrine effects in the aquatic environment are in the focus of scientists and media along with debates on the necessity of further steps in wastewater treatment. In the present study VTG responses were compared to evaluate upgrades at wastewater treatment plants (WWTPs). We investigated several advanced sewage treatment technologies at two WWTPs connected to the Schussen, a tributary of Lake Constance, for the reduction of hormonal activity: (1) a powdered activated charcoal filter at the WWTP Langwiese; and (2) a combination of ozonation, sand filter, and granulated activated carbon filter at the WWTP Eriskirch. Rainbow trout and brown trout were either directly exposed to the effluents in aquaria or cages, or in a bypass system flown through by surface water of the Schussen. As a reference, trout were kept in bypass aquaria at the Argen River, which is less influenced by micropollutants. As a biomarker for estrogenicity, we analyzed the yolk precursor protein vitellogenin in immature rainbow trout and brown trout and in trout larvae (100 days post-fertilization) prior to and after the upgrade with the new technologies. Trout of different ages and species were used to detect differences in their sensitivity. At both bypass stations, larvae of brown trout showed significantly higher vitellogenin levels prior to the upgrade compared to negative control levels. Female brown trout exposed at the bypass station downstream of the WWTP showed decreased vitellogenin levels after the upgrade. In 1-year-old immature trout directly exposed to the respective effluents, no significant effects of the upgrades on vitellogenin levels were found. In general, larger effects were observed in brown trout than in rainbow trout, indicating that they are more sensitive test organisms.

## Background

Endocrine disruptors (EDs) are hormonally active chemicals which are able to influence the endocrine system of organisms by mimicking or repressing the body’s own hormones. EDs are a very diverse group of chemicals including, for example, ingredients of personal care products, pharmaceuticals containing steroid hormones, pesticides, plasticizers, dioxins, furans, phenols, alkylphenols, polychlorinated biphenyls, and brominated flame retardants [1, 2]. Still more endocrine-active chemicals were identified over the last years. The priority list of the European Commission contains 564 chemicals that had been suggested by various organizations and published papers as being suspected EDs [1].

Because the aquatic environment is an important sink for natural and anthropogenic chemicals [3], the release of pollutants including EDs into surface waters via wastewater treatment plants (WWTPs) has come into the focus of scientists, authorities, and the public. Today, most wastewater is treated before it is released into bodies of water, but many studies show that not all hazardous chemicals, especially EDs, can be completely removed by routine wastewater treatment (see, e.g., [4]). Therefore, the discharge of wastewater treatment plants into recipient rivers is a main source for EDs to enter the aquatic environment. The level of pollution in rivers is particularly high if the catchment area is highly populated, has industry, or agriculture. Because wastewater can contribute up to 50 % and more of the flow of a river in months with low water [3], the released chemicals can play an important role for the occurring biota. For example, steroid estrogens, like the pharmaceutical ethinyl estradiol (EE2), are known to be extraordinarily active in fish at low to sub-ng/L concentrations [5, 6], and are found in many WWTP effluents at effect concentrations [7, 8].

This raises the question whether we should eliminate more pollutants, especially EDs, to improve wastewater quality. Whereas, for example, the Swiss Federal Government started projects introducing a tertiary treatment step at many of its WWTPs, the discussion whether additional wastewater treatment technologies are ecologically worthwhile is still ongoing [9].

**Fig. 1:**
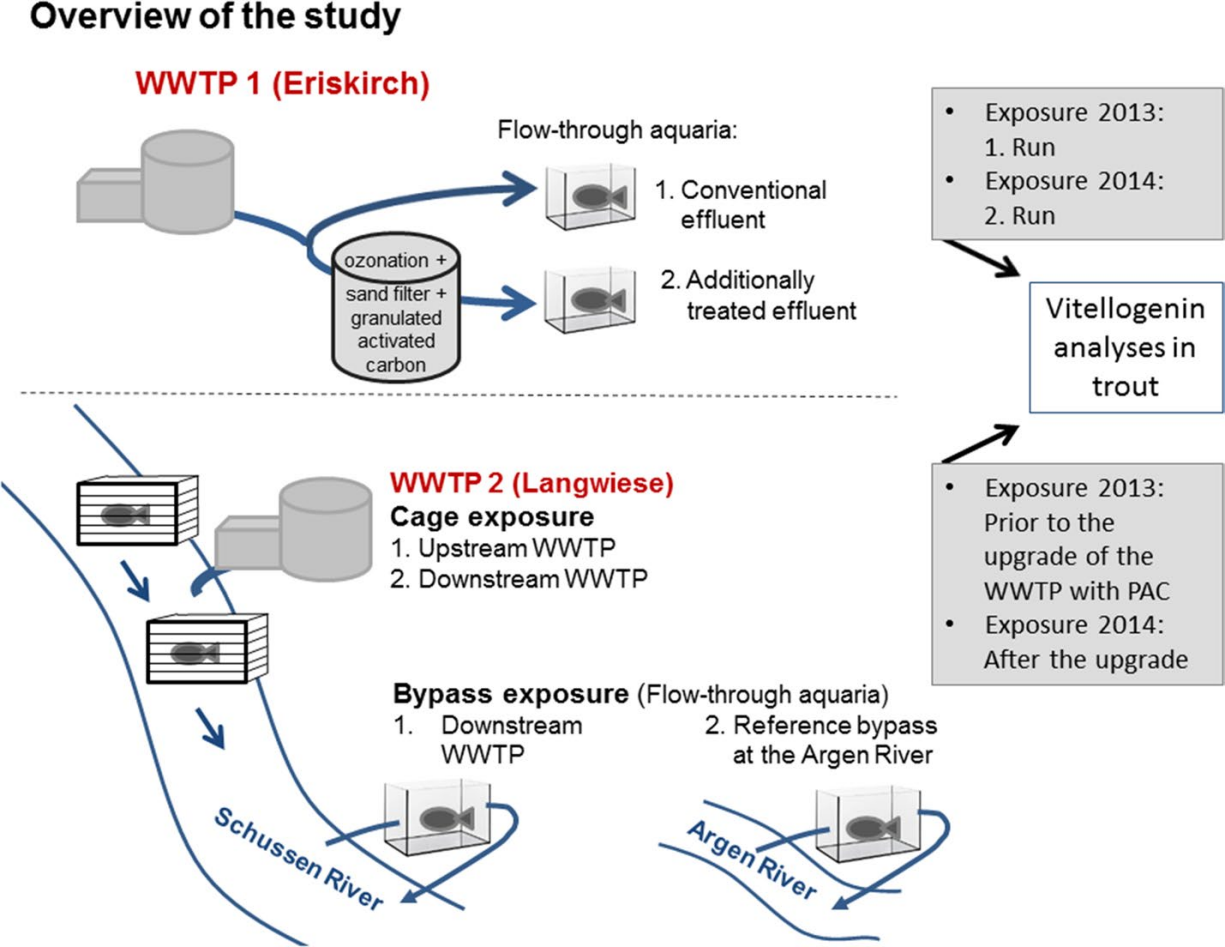
Overview of the study design. *PAC* powdered activated charcoal

The present study is part of the “SchussenAktiv*plus*” project in the Lake Constance area investigating differently sized WWTPs which were equipped with additional wastewater treatment techniques [10]. Two of them (WWTP Langwiese and WWTP Eriskirch) are in the focus of the present study. To characterize the efficiency of technologies newly introduced at these WWTPs, we investigated vitellogenin (VTG) in juvenile male and female trout as well as in trout larvae as a biomarker of estrogenicity [5, 11–16]. VTG is an egg yolk precursor protein which is normally only produced by female fish. It is estrogen-dependent and EDs can act on hepatic receptors to induce the synthesis of VTG in males and juveniles [11, 17]. We compared VTG levels of trout that were exposed (1) directly to the conventional and modified effluent in aquaria connected to the effluents; (2) upstream and downstream the effluent prior and after the WWTP upgrade; and (3) in bypass systems downstream the WWTP and at a reference river prior and after the WWTP upgrade. Figure 1 gives an overview of these three approaches. It also shows the two WWTPs with their new technologies and summarizes the exposure experiments in the years 2013 and 2014 (for detailed information see methods section).

## Results and discussion

### Exposure experiments at the WWTPs

**Fig. 2:**
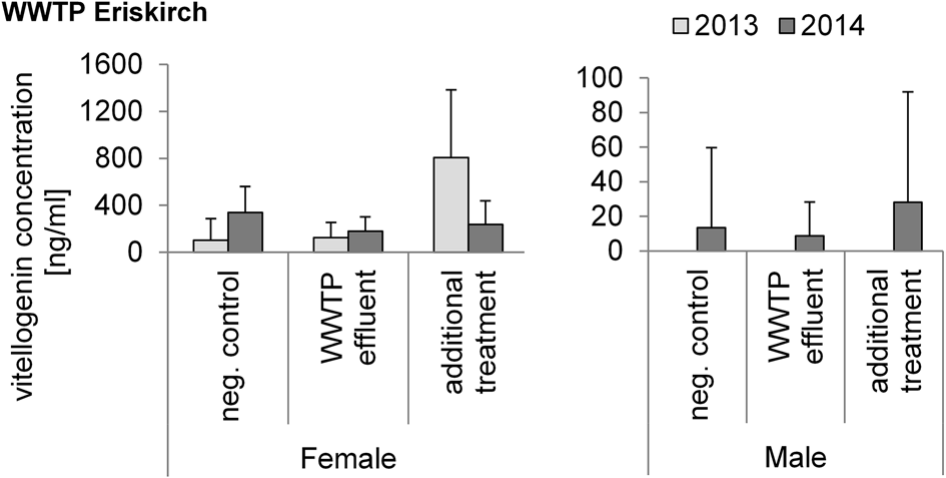
Vitellogenin concentrations in blood plasma samples of rainbow trout exposed at the WWTP Eriskirch in aquaria connected to the conventional effluent or to the additionally treated effluent in 2013 and 2014; means and standard deviation (SD) are shown. Analyzed by Biosense rainbow trout vitellogenin ELISA kit. *N*-numbers 2013 females: negative control *n* = 9, WWTP effluent *n* = 6, additional treatment *n* = 8; males: negative control *n* = 1, WWTP effluent *n* = 7, additional treatment *n* = 3. No significant differences with Steel–Dwass test; *p* > 0.05. *N*-numbers 2014 females: negative control *n* = 6, WWTP effluent *n* = 5, additional treatment *n* = 4; males: negative control *n* = 13, WWTP effluent *n* = 6, additional treatment *n* = 10. No significant differences with Steel–Dwass test; *p* > 0.05. No significant differences between years; *p* > 0.05

In rainbow trout exposed at the conventional and modified effluent at the WWTP Eriskirch, VTG levels in females varied between treatments (conventional and additionally treated effluent) and years (exposure in 2013 and 2014), whereas VTG levels in males were constantly low or even non-detectable (significant differences could not be determined) in both years (Fig. 2).

The increased VTG level in females in 2013 after the exposure to additionally treated wastewater might be due to the altered composition of the effluent in 2013 compared to 2014 with more ozone used in 2013 compared to 2014. This possibly could have resulted in the formation of by-products with estrogenic activity [18]—thus leading to higher VTG levels in females. The lacking reactions in male fish, however, indicate that the wastewater at the WWTP Eriskirch was not highly estrogenic in general. In line with this, chemical analyses of the effluent showed only low concentrations of estrogen-active substances (Bisphenol A: 39–110 ng/L in the conventional effluent and 11-160 ng/L after the additional treatment) or concentrations below the detection limit (EE2 > 1 ng/L) [19].

In contrast to our results, a study with crucian carp showed that VTG levels in immature female and male carps were reduced when the wastewater was treated with ozone [20]; however, the carps already had higher VTG induction in the normal effluent compared with controls and our trout did not show higher VTG levels in the normal effluent compared with the negative controls.

**Fig. 3:**
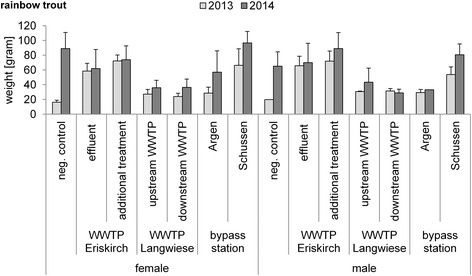
Means of weight (gram) and SD of exposed rainbow trout in 2013 and 2014. For *n*-numbers see Table 1 and for significant differences see Table 2

**Fig. 4:**
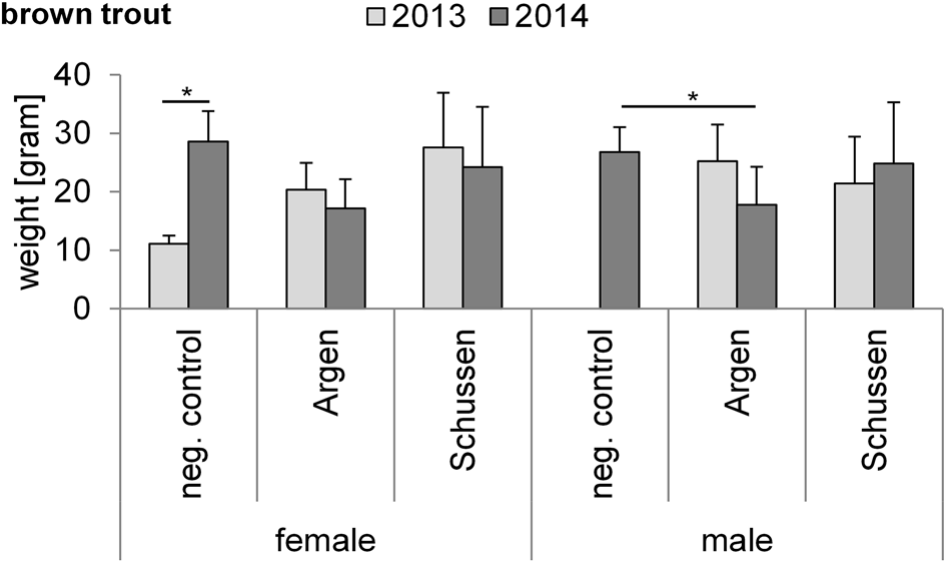
Means of weight (gram) and SD of exposed brown trout in 2013 and 2014. For *n*-numbers see Table 3. Significant differences with the Tukey–Kramer HSD test: females: neg. control 2013—neg. control 2014 *p* = 0.0226 and males 2014: neg. control—Argen *p* = 0.0498 (*Asterisks* significant differences; *p < 0.05)

Furthermore, we observed slightly higher VTG levels in females of our negative control in 2014 compared to 2013. Differences in VTG baseline levels in negative controls between the years 2013 and 2014 were probably due to the slower fish growth in the laboratory in 2013. In 2013, the mean weight of rainbow trout was 16.3 g ± 2.7 SD and, in 2014, the mean weight was 89 g ± 21.8 SD (Fig. 3). The brown trout showed similar results (Fig. 4). The gonadal development depends on the size of a fish. The bigger the fish the more developed are its gonads, and developed gonads are associated with higher VTG concentrations because the gonads induce the VTG synthesis in liver cells via hormones [21].

**Table 1:** *N*-numbers of exposed rainbow trout in 2013 and 2014

Year	Treatment	*N*-numbers	
		Female	Male
Rainbow trout			
2013	Neg. control	9	1
WWTP Eriskirch	Effluent	6	7
	Additional	8	3
WWTP Langwiese	Upstream WWTP	15	2
	Downstream WWTP	7	4
Bypass	Argen	8	5
	Schussen	4	9
2014	Neg. control	6	13
WWTP Eriskirch	Effluent	5	6
	Additional	4	10
WWTP Langwiese	Upstream WWTP	9	11
	Downstream WWTP	13	8
Bypass	Argen	8	1
	Schussen	6	16

**Table 2:** Significant differences in weights of rainbow trout

Year	Treatment group	*p* value
Females		
2013	Neg. control 2013—additional treatment WWTP Eriskirch 2013	0.041
	Neg. control 2013—upstream WWTP Langwiese 2013	0.0080
2014	Downstream WWTP Langwiese 2014—bypass Schussen 2014	0.0465
2013 vs 2014	Neg. control 2013—upstream WWTP Langwiese 2014	0.0372
	Neg. control 2013—downstream WWTP Langwiese 2014	0.0081
	Upstream WWTP Langwiese 2013—additional treatment WWTP Eriskirch 2014	0.0092
	Upstream WWTP Langwiese 2013—effluent WWTP Eriskirch 2014	0.0451
	Upstream WWTP Langwiese 2013—neg. control 2014	0.035
	Upstream WWTP Langwiese 2013—bypass Schussen 2014	0.035
	Neg. control 2013—bypass Argen 2014	0.041
	Additional treatment WWTP Eriskirch 2013—upstream WWTP Langwiese 2014	0.0407
	Additional treatment WWTP Eriskirch 2013—downstream WWTP Langwiese 2014	0.0139
Males		
2014	Neg. control 2014—downstream WWTP Langwiese 2014	0.0138
	Additional treatment WWTP Eriskirch 2014—upstream WWTP Langwiese 2014	0.0411
	Additional treatment WWTP Eriskirch 2014—downstream WWTP Langwiese 2014	0.0299
	Bypass Schussen 2014—downstream WWTP Langwiese 2014	0.0076
	Bypass Schussen 2014—upstream WWTP Langwiese 2014	0.0201
2013 vs 2014	Bypass Schussen 2013—bypass Schussen 2014	0.0331

Data were logarithmised to get homoscedastic data and the Steel–Dwass test revealed the following *p* values

**Table 3:** *N*-numbers of exposed brown trout in 2013 and 2014

Year	Treatment	*N*-numbers	
		Female	Male
Brown trout			
2013	Neg. control	4	–
	Argen	4	4
	Schussen	3	4
2014	Neg. control	6	10
	Argen	6	13
	Schussen	9	5

**Fig. 5:**
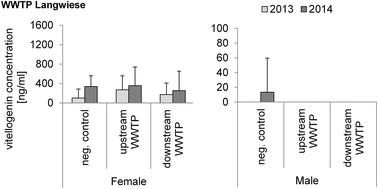
Vitellogenin concentrations in blood plasma samples of rainbow trout exposed in 2013 and 2014 in cages upstream and downstream of the WWTP Langwiese; means and SD are shown. Analyzed with Biosense rainbow trout vitellogenin ELISA kit. *N*-numbers 2013 females: negative control *n* = 9, upstream WWTP *n* = 15, downstream WWTP *n* = 7; males: negative control *n* = 1, upstream WWTP *n* = 2, downstream WWTP *n* = 4. No significant differences with Steel–Dwass test; p > 0.05. *N*-numbers 2014 females: negative control *n* = 6, upstream WWTP *n* = 9, downstream WWTP *n* = 13; males: negative control *n* = 13, upstream WWTP *n* = 11, downstream WWTP *n* = 8. No significant differences with Steel–Dwass test; *p* > 0.05. No significant differences between years; *p* > 0.05

The results of the caging experiments performed upstream and downstream of the WWTP Langwiese showed no evidence of estrogenic disruption in males, neither before nor after the upgrade (Fig. 5). Chemical analyses found no EE2 in the effluent (detection limit 1 ng/L), but in vitro tests revealed estrogenic potentials prior to the upgrade [22]. In females, slightly, but not significantly higher VTG levels were measured upstream the WWTP in both years. Lower values downstream might possibly be caused by the combined activity of estrogenicity, anti-estrogenicity, and androgenicity which were all detected in parallel in in vitro bio tests [22].

In summary, the results of our exposure experiments at the two WWTP effluents made evident that, in contrast to other studies which showed an induction of VTG by wastewater in juvenile, sexually immature, and male trout [11, 17, 23], even the conventional effluents of these WWTPs did not lead to increased VTG levels. This speaks for the high efficiency of the already established technologies at these two WWTPs, which, like most of the other larger WWTPs connected to tributaries of Lake Constance, are already equipped with a flocculation sand filter as a final cleaning step.

### Exposure experiments at the bypass stations at the Schussen and the Argen River

#### Rainbow trout

**Fig. 6:**
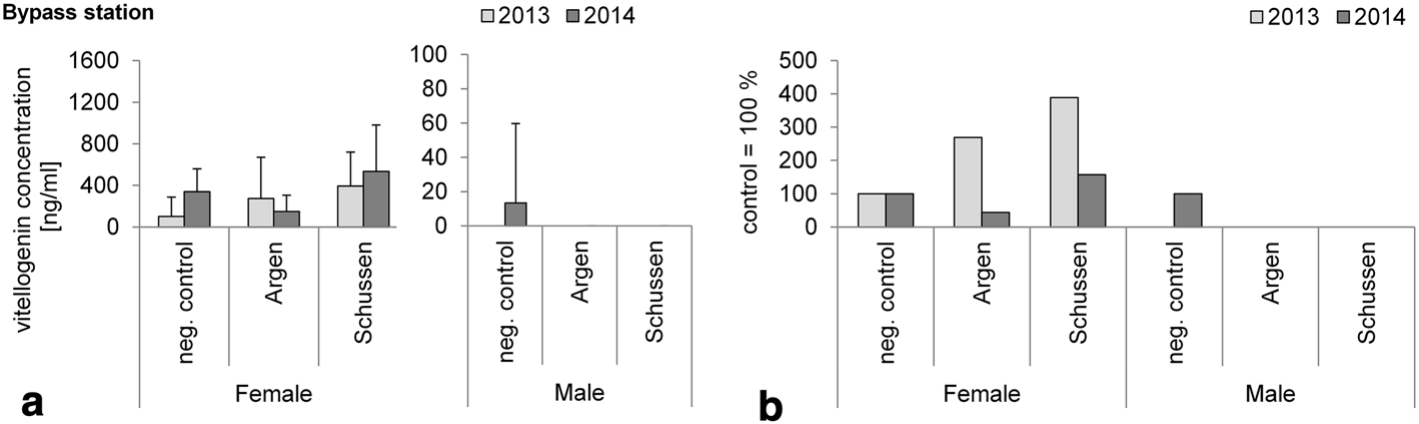
**a** Vitellogenin concentrations in blood plasma samples of rainbow trout exposed in 2013 and 2014 at the bypass stations; means and SD are shown. Analyzed by Biosense rainbow trout vitellogenin ELISA kit. *N*-numbers 2013 females: negative control *n* = 9, Argen *n* = 8, Schussen *n* = 4; males: negative control *n* = 1, Argen *n* = 5, Schussen *n* = 9. No significant differences with Steel–Dwass test; *p* > 0.05. *N*-numbers 2014 females: negative control *n* = 6, Argen *n* = 8, Schussen *n* = 6; males: negative control *n* = 13, Argen *n* = 1, Schussen *n* = 16. No significant differences with Steel–Dwass test; *p* > 0.05. No significant differences between years; *p* > 0.05. **b** Values of **a** relative to negative control. Neg. control was set to 100 %

In the two bypass systems, at the Schussen downstream the WWTP Langwiese and at the reference river Argen, no VTG induction became evident in male fish, neither before nor after the upgrade of the WWTP Langwiese with the powdered activated charcoal filter (Fig. 6a). The VTG levels in females were highly variable; however, the highest percentages production in relation to the levels in the respective negative control fish were found in trout exposed at the bypass at the Schussen prior to the WWTP upgrade (Fig. 6b). Significant differences, however, did not occur.

Juvenile rainbow trout which hatched at the bypass stations and were continuously exposed there afterwards showed neither before nor after the upgrade any induction of VTG. In contrast to that, Stalter et al. showed a significant increase in the VTG concentrations using yolk sac rainbow trout which were directly exposed to WWTP effluents for 60 days [17]. We used river water instead of effluent and the results of our other experiments revealed only a weak estrogenic pollution, explaining why we did not find increased VTG levels in juveniles.

These results for juvenile rainbow trout coincide with data for male fish, both indicating that neither at the Schussen downstream the WWTP nor at the Argen River are rainbow trout affected by estrogen disruptors.

#### Brown trout

**Fig. 7:**
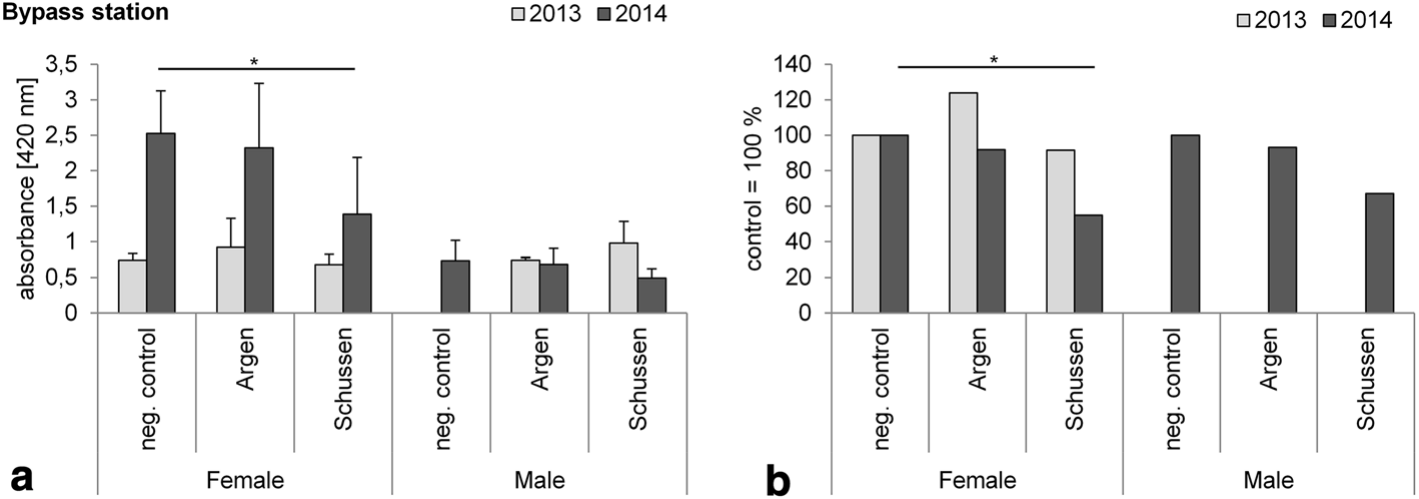
**a** Absorbance measured in blood plasma samples of 1-year-old brown trout exposed at the bypass stations in 2013 and 2014; means and SD are shown. All samples of a group were analyzed within one semi-quantitative vitellogenin salmonid (Salmoniformes) biomarker ELISA kit (enzyme activity = color intensity is proportional to the concentration of vitellogenin in the sample). *N*-numbers 2013 females: negative control *n* = 4, Argen *n* = 4, Schussen *n* = 3; males: negative control *n* = 0, Argen *n* = 4, Schussen *n* = 4. No significant differences; *p* > 0.05. *N*-numbers 2014 females: negative control *n* = 6, Argen *n* = 6, Schussen *n* = 9; males: negative control *n* = 10, Argen *n* = 13, Schussen *n* = 5. Significant differences with the Tukey–Kramer HSD test: females 2014 neg. control—Schussen p = 0.0231 (*Asterisks* significant differences; *p < 0.05). **b** Values of **a** relative to negative control. Neg. control was set to 100 %. In 2013, no values could be given for males because of absence of males in the neg. control

In 2013, prior to the upgrade, we found no significant differences in VTG levels in female and male brown trout exposed at the bypass stations (Fig. 7a). In 2014, after the upgrade, female brown trout showed significantly lower VTG levels at the Schussen (downstream WWTP Langwiese), whereas males showed no significant differences (Fig. 7a). Note that VTG levels of brown trout from different years cannot be compared because semi-quantitative VTG kits (semi-quantitative Salmonid (*Salmoniformes*) biomarker ELISA from Biosense) were used, implying that values are only comparable within one kit. This is the reason why the absolute values are also presented as relative values to the respective negative control levels in Fig. 7b. In 2014, VTG levels of females and males were lower at both rivers compared to negative control levels. Fish size did not vary strongly within treatment groups of each year (Fig. 4). Especially females showed no significant differences in their weights in 2014, hence we excluded differences in size as an explanation for differences in VTG levels (see Fig. 4). The fact that the VTG levels in females exposed at the Schussen were significantly lower than the negative control might be explained by the upgrade of the WWTP Langwiese. The additional treatment step at the WWTP Langwiese might have reduced estrogenic activities, and thereby unmasked anti-estrogenic activities which led to reduced VTG levels. Results by Stalter et al. indicated the importance of masking effects to evaluate wastewater [24]. Analyses of the same samples by in vitro yeast assays provided supporting results by showing elevated anti-estrogenicity and degraded estrogenic activities after the upgrade (not published).

**Fig. 8:**
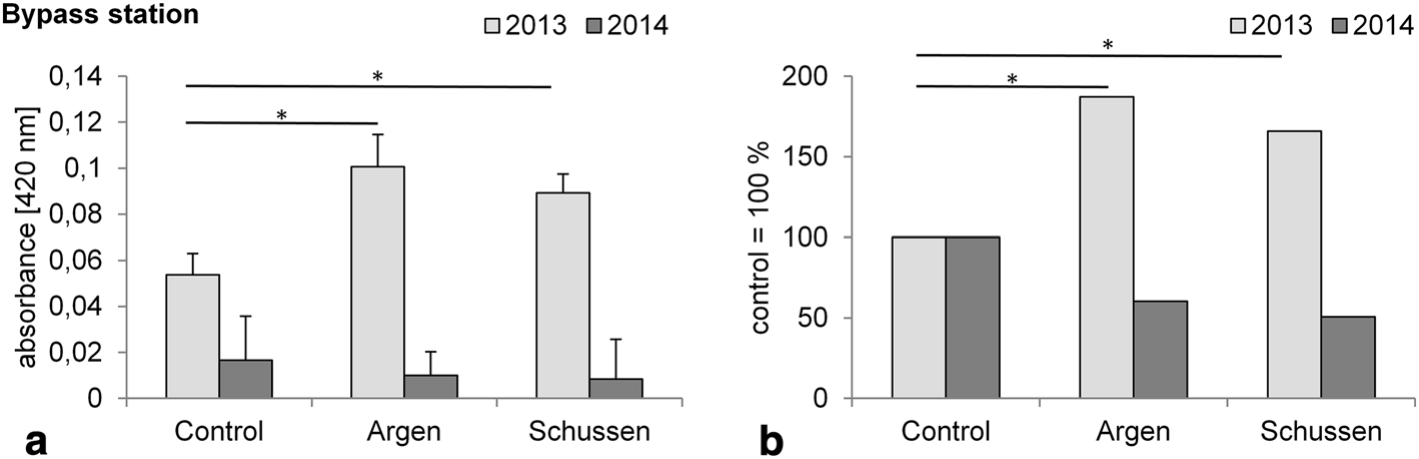
**a** Absorbance measured in homogenates of juvenile brown trout exposed for 99 days post-fertilization at the bypass stations in 2014; means and SD are shown. All samples were analyzed within one semi-quantitative vitellogenin salmonid (Salmoniformes) biomarker ELISA kit (enzyme activity = color intensity is proportional to the concentration of vitellogenin in the sample). Each treatment *n* = 12. No significant differences with the Steel–Dwass test; *p* > 0.05. For better comparison, previous results from 2013 prior to the upgrade are also shown. These results were already published in PlosOne by Henneberg et al. 2014 [22]. **b** Values of **a** relative to negative control. Neg. control was set to 100 %

At both bypass stations, brown trout larvae showed no increased VTG values after the upgrade of the WWTP Langwiese compared to the negative control levels (Fig. 8a). On the contrary, the levels are even lower than the negative control levels, which might again be related to unmasked anti-estrogenicity in 2014. These results differ from data we collected prior to the upgrade (Fig. 8, and see also Henneberg et al. [22]). In this previous study, brown trout showed significantly higher VTG levels at the Schussen bypass and at the Argen bypass compared to the negative control after the same exposure time. Estrogen-active compounds were likely causes for the increased VTG levels prior to the upgrade. Due to the fact that we did not observe differences in VTG levels after the upgrade at both bypass stations, we conjecture that it is mainly annual specific differences that caused these effects, and to a lesser degree the upgrade itself.

In contrast to these results for brown trout, we observed no differences in VTG levels of juvenile and 1-year-old rainbow trout. Previous studies showed that brown trout are more sensitive to environmental stress than rainbow trout [25–27], and our results are in line with this observation. Bjerregaard et al. concluded that the sensitivity of brown trout to estrogens does not differ from the sensitivity of the majority of fish species; first- and second-year brown trout appear to be suitable monitoring organisms to demonstrate estrogenic effects in headwater streams [28]. Hence, our results indicate slight temporary estrogenic effects that might affect feral fish species. However, the differences in VTG levels of brown trout we observed were low, and we conclude that estrogenic effects in the two rivers investigated are generally low.

Organs of the trout we used in the present study were examined in a parallel study to assess their health status before and after the upgrade at the WWTP Langwiese. The results showed that the upgrade led to a better health status of trout and partly also of feral fish species. While this showed that the upgrade reduced toxic effects, the current study showed that estrogenic effects were only slightly reduced.

## Conclusion

Overall, our VTG results showed no strong estrogenic effects of WWTP effluents at the Schussen River on trout. After the upgrade of WWTP Langwiese, juvenile and female brown trout showed significantly decreased VTG levels but especially the results for brown trout larvae indicated that annual variation might also play a major role. While rainbow trout showed no significant reduction in VTG levels, we found reduced VTG levels in brown trout, indicating that brown trout might respond more sensitively than rainbow trout.

Furthermore, we did not observe increased VTG levels in males in any experiment. Therefore, we classify the Schussen River as showing only low pollution with estrogens. In particular, neither effluents of the WWTP Langwiese nor effluents of the WWTP Eriskirch caused significantly higher VTG levels in trout, independently of additional wastewater treatment technologies.

## Methods

### Test organisms

For our investigations we used immature, 1-year-old brown trout (*Salmo trutta* f. *fario*) and rainbow trout (*Oncorhynchus mykiss*) delivered by the fish hatchery Lohmühle, Alpirsbach, Germany. We also obtained freshly fertilized trout eggs from there. For the experiments trout were transported from the hatchery to the exposure sites and directly released in cages or aquaria. The trout which grew up at the fish farm received a mixture of spring water with drinking water quality and stream water which originates in a water protection area (pH 7, nitrate <0.3 mg/L, nitrite <0.0033 mg/L) [29]. All fish were fed with food from the company BioMar, Denmark (INICO Plus for larvae and EFICO alpha for 1-year-old trout) in different particle sizes, depending on fish size. Trout in all our exposure experiments received the same amount of food, except for the negative control (rainbow trout) in 2014, which were sampled directly at the fish farm and for which the amount fed was not under our control.

### Exposure experiments at WWTPs and at bypass systems

**Fig. 9:**
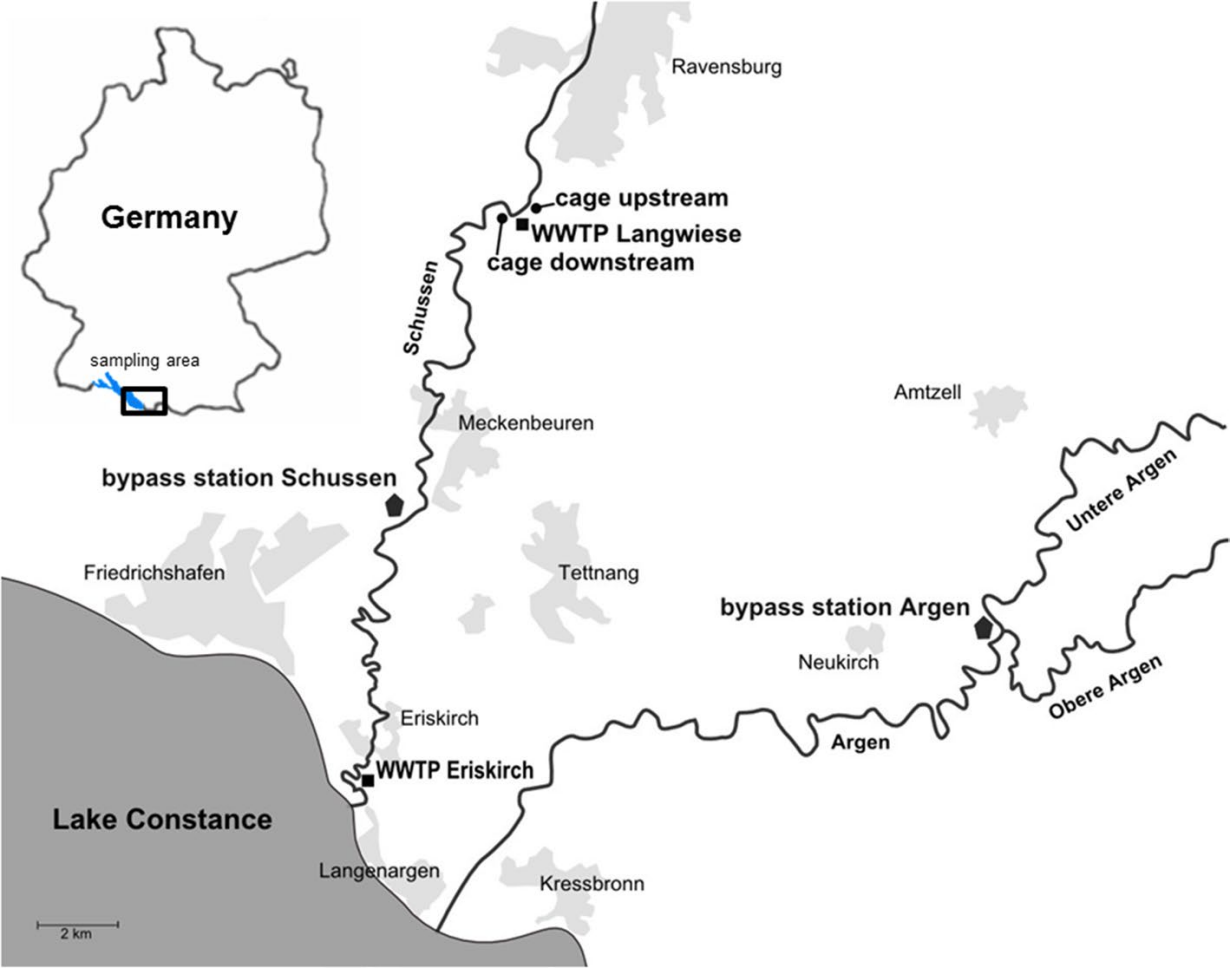
Overview of sampling sites, bypass stations and examined WWTPs at the Schussen River and Argen River, Lake Constance, South Germany

As a model for a medium-sized WWTP with 40,000 population equivalents we chose the WWTP Eriskirch connected to the Schussen River in the Lake Constance catchment area, South Germany (Fig. 9). At this WWTP, a small-scale model installation was realized in 2013, which included different columns allowing cleaning of partial effluent flow by different combinations of ozonation, sand filtration, and granulated activated carbon filter. In 2013 and 2014, 1-year-old rainbow trout were exposed here in aquaria of which one was flown through by the conventional effluent and the second by the additionally treated effluent. In 2013, the additionally treated effluent was proportionately composed of wastewater treated by (1) ozonation + sand filter + granulated activated carbon, and (2) ozonation + granulated activated carbon. In 2014, the composition was changed as follows: (1) ozonation + sand filter; (2) ozonation + granulated activated carbon; and (3) only granulated activated carbon in the ratio 1:1:1. The aquarium with the regular effluent was aerated via a membrane pump to ensure sufficient oxygen concentrations for trout. Daylight was simulated by lamps using timer clocks, and the light/dark photoperiod was adapted to natural daylight. Fish were fed with equal amounts of food by an automatic feeder once a day.

As a model for a large WWTP with 170,000 population equivalents, the WWTP Langwiese was in the focus of our study, also situated at the Schussen River upstream of the WWTP Eriskirch (Fig. 8). At the WWTP Langwiese, an additional powdered activated carbon filter was put into operation after the biological treatment and before the final sand filter in September 2013. At that WWTP, we exposed 1-year-old rainbow trout in cages (for cage description see [30]) 100 m upstream the WWTP effluent and downstream of it (mixture of 50 % effluent and 50 % Schussen water) in the Schussen River. Trout were fed every second day with a comparable amount of food as the trout at the WWTP Eriskirch received in 2 days. The exposure experiments at the WWTP Langwiese were performed in spring 2013 prior to the upgrade with the powdered activated carbon filter and in spring 2014 after the upgrade.

In addition to the exposure experiments at the WWTPs, we used two bypass stations with 250 L aquaria continuously flown through by fresh river water (0.4 L/s): one setup was located downstream of the WWTP Langwiese at the Schussen River and one at the Argen River as a reference river less influenced by micropollutants [31] (see Fig. 9). Here, fertilized eggs and developing larvae as well as 1-year-old brown trout and rainbow trout were exposed (for a detailed description of the bypass station and trout exposure conditions see [22]).

As a negative control, we kept trout in 250 L aquaria under semi-flow-through conditions in climate chambers at the University of Tübingen. We used filtered tap water and exchanged a third of the water volume once a week. Water was aerated, temperature was kept at 6 °C, a stream pump (Co.: Tunze, Germany) guaranteed a constant stream, and a filter (Co.: JBL1500e) kept good water conditions. Temperature, ammonium and nitrite concentrations were controlled every other day (ammonium <0.05 mg/L, nitrite <0.01–0.05 mg/L). Light/dark photoperiod was adapted to natural daylight. The semi-static conditions implied that we could not feed fish as much as in the flow-through systems because we had to keep a good water quality. The poor growth of our negative control fish in 2013 was a main reason for us to change the negative control fish in 2014. For that, we sampled 1-year-old trout in 2014 directly at the fish farm where we bought all our trout. To ensure that the development status in all groups was comparable, we sampled the negative control fish at the fish farm shortly before sampling fish at the WWTPs.

To ensure that fish generally react to estrogenic substances by producing VTG, we exposed trout to EE_2_ as a positive control. For this, fish were kept at same conditions as negative control fish in 2013, but EE_2_ was added in concentrations which ranged from 5 to 20 ng/L. All trout exposed to EE_2_ showed extreme higher VTG levels than the negative controls (see Table 4).

**Table 4:** Mean values and SD of exposure experiments with trout using EE_2_ as positive control

	Females		Males	
	2013	2014	2013	2014
Brown trout				
Mean values (absorbance [420 nm])	326.33	21.77	500.5	4.86
SD	±70.11	±25.09	±114	±3.1
*n*-number	3	5	2	5
Rainbow trout				
Mean values (VTG [ng/ml])	2,699,183.9	3,812,659.5	3,362,476.1	3,830,203.9
SD	±3,074,723.7	±1,653,878.4		±1,867,237.4
*n*-number	8	5	1	7

Juvenile trout	Rainbow trout (VTG [ng/ml])	Brown trout (absorbance [420 nm])
Mean values	2,030.54	0.0808
SD	±2811.60	±0.0358
*n*-number	8	9

### Exposure duration at WWTPs and at bypass systems

Prior to the upgrade at the WWTP Langwiese, we carried out one bypass exposure and one cage exposure experiment in the winter season 2012/2013. After the upgrade at the WWTP Langwiese, one bypass exposure and one cage exposure experiment were performed in the winter season 2013/2014. At the WWTP Eriskirch, we started the first exposure experiment in spring 2013 because the installation of the exposure aquaria was not completed until then. In the second year, 2014 (after the upgrade of the WWTP Langwiese), all exposure experiments started at the same time at all sites. Tables 5 and 6 summarize the time schedule for all exposure experiments, including exposure duration and exposure type.

**Table 5:** Time schedule for the exposure experiments performed at WWTPs and bypass stations with 1-year-old trout

Start of exposure	End of exposure	Exposure duration (days)	Exposure type	Trout species
Winter season 2012/2013 prior to the upgrade				
15 Nov 2012	24 Jan 2013	70	Laboratory neg. control + EE_2_ control	Brown and rainbow trout
15 Nov 2012	17 Jan 2013	63	Cage exposure	Rainbow trout
15 Nov 2012	14 Feb 2013	91	Exposure in bypass systems	Brown and rainbow trout
6 Feb 2013	21 Mar 2013	43	Exposure at WWTP Eriskirch	Rainbow trout
Winter season 2013/2014 after the upgrade				
	29 Jan 2014	0	Neg. control from hatchery	Brown and rainbow trout
2 Dec 2013	23 Jan 2014	52	EE_2_ control	Brown and rainbow trout
2 Dec 2013	4 Feb 2014	64	Cage exposure	Rainbow trout
2 Dec 2013	13 Feb 2014	73	Exposure at WWTP Eriskirch	Rainbow trout
2 Dec 2013	12 Mar 2014	100	Exposure in bypass systems	Brown and rainbow trout

**Table 6:** Time schedule for exposure experiments performed at the bypass stations with fresh fertilized trout eggs

Start of exposure	End of exposure	Exposure duration	Exposure type	Trout species
Winter season 2012/2013 prior to the upgrade				
07 Dec 2012	20 Mar 2013	103 days	Laboratory neg. control	Rainbow trout
07 Dec 2012	21 Mar 2013	104 days	Exposure in bypass systems	Rainbow trout
Results of exposure experiments using juvenile brown trout are published in Henneberg et al. [22].				
Winter season 2013/2014 after the upgrade				
24 Nov 2013	3 Mar 2014	99 days	Laboratory neg. control	Brown and rainbow trout
24 Nov 2013	4 Mar 2014	100 days	Exposure in bypass systems	Brown and rainbow trout
7 Mar 2014	28 Mar 2014	22 days	EE_2_ control	Brown and rainbow trout

### Ethic statement

This study was carried out in strict accordance with German legislation (animal experiment permit nos. ZO 1/09 and ZP 1/12, District Magistracy of the State of Baden-Württemberg).

### Vitellogenin detection

#### Sampling

One-year-old brown trout and rainbow trout, sampled at each site, were killed with an overdose MS-222 (tricaine mesylate, Sigma-Aldrich, St. Louis, USA). Blood samples were taken immediately from the caudal vein by a sterile syringe, transferred in lithium-heparinized reaction tubes (Co. Sarstedt, Germany), and 4 TIU aprotinin (C. Roth, Germany) per mL blood was added. Samples were centrifuged (4 °C, 10 min, 2500 rpm Eppendorf 5810R) on-site and plasma samples were snap-frozen in liquid nitrogen. Thereafter, plasma aliquots were stored at −80 °C until we determined VTG levels. After taking the blood samples, the length and weight of each fish were measured, gonads were removed for histological examinations and fixed in 2 % glutaraldehyde dissolved in 0.1 M cacodylic acid.

Larvae were killed with an overdose MS-222 (tricaine mesylate, Sigma-Aldrich, St. Louis, USA), and the region between head and pectoral fin from each individual was placed in Eppendorf tubes, snap-frozen, and stored at −80 °C.

All the following steps were undertaken on ice. Homogenates of juvenile trout were prepared by adding homogenization buffer (4 times the sample weight; PBS + 2 TIU aprotinin, C. Roth, Germany), mixing with a plastic pestle, centrifuging (10 min, 4 °C, 20,000×*g* Eppendorf 5810R) [17] and storing the supernatants at −80 °C.

#### Vitellogenin ELISA

VTG levels of rainbow trout were measured using the rainbow trout (*Oncorhynchus mykiss*) vitellogenin ELISA kit (V01004402, Biosense Laboratories, Norway). For the analyses of the brown trout samples we used a semi-quantitative kit because the antibody of this kit shows a very good cross-reactivity against brown trout VTG (semi-quantitative vitellogenin Salmonid (*Salmoniformes*) biomarker ELISA kit (V01002402, Biosense Laboratories, Norway)). All steps were performed as described in the protocols. As recommended by the provider of the test kit, a minimum of 1:20 dilution was used and samples were tested in duplicates. The absorbance was measured by a microplate reader (Automated Microplate Reader Elx 8006, Bio-Tek Instruments, INC., USA).

The semi-quantitative ELISA test kit, which is recommended for VTG analyses of salmonids, was used for our brown trout samples. The enzyme activity (absorbance), which is measured by the assay, is proportional to the concentration of VTG in the sample. Purified VTG from Atlantic salmon (*Salmo salar*) was used as a positive control within every assay run. We analyzed all blood samples of females with one 96-well plate (neg. control, Bypass Schussen, Bypass Argen and, EE2 control), all samples of males on the next 96-well plate, etc. Hence, all these samples are comparable within their groups. All steps were performed as described in the protocols by the provider of the test kit.

### Statistical analyses

Statistical analyses were performed with JMP 10.0 (SAS Systems, USA). Data were tested for normality using the Shapiro–Wilk W test and for homogeneity of variance with the Levene test. If the data were normally distributed and the variance was homogeneous, the Tukey–Kramer HSD test was conducted. Otherwise, if the data were homoscedastic but not normally distributed, the Steel–Dwass test was used. If the data were normally distributed but not homoscedastic, the Welch’s ANOVA was performed.
